# M. Beaupré on the Historical and Medical Effects of Cold in the Russian Campaign

**Published:** 1826-07

**Authors:** 


*A Treatise on the Effects and Properties of Cold, with a Sketch, Historical and Medical, of the Russian Campaign.* By Moricheau Beaupré, M.D. Regimental Surgeon in the French Service. Translated by John Clendinning, A.B. and M.D. with an Appendix by the Translator. Octavo, pp. 375. Edinburgh, Maclachlan and Stewart, &c. 1826.

---

"——— fluentemque tabem liquescentis nivis ingrediebantur. Tetra ibi luctatio erat. Ut a lubrica glacie, non recipiente vestigium, et in prono citius pede se fallente, et, seu manibus in adsurgendo seu genu se adjuvissent, ipsis adminiculis prolapsis, iterum corruissent, nec stirpes circa radicesve, ad quas pede aut manu quisquam eniti posset, erant; ita in levi tantum glacie tabidaque nive volutabantur. Jumenta secabant; interdum etiam, tum infimam ingredientia nivem, et prolapsa jactandis gravius in continendo ungulis, penitus perfringebant: ut pleraque, velut pedica capta, hærerent in durata et alte concreta glacie."—*Livii Histor. Lib.* xxi. *Cap.* xxxvi.

---

The motto we have chosen exhibits a frightful picture of the miseries which Hannibal and his soldiers endured in their passage across the Alps, and these had scarcely any parallel in ancient or modern history when Napoleon, at the head of a powerful army, and flushed with conquest, thoughtlessly encountered all the dangers and horrors of a Russian winter.

We learn from Quintus Curtius* that Alexander the Great and his brave followers laboured under almost incredible hardships from cold, not only amid snows in the barbarous regions of Northern Asia, but when they passed the Tanais to subjugate the Scythians. These unfortunate warriors, overcome with necessary wants, sank into despair, and numbers either perished on the road, or lost their feet by congelation. Margat mentions that Tamerlane, accompanied by his numerous hordes of Tartars, and while advancing into Russia, was overtaken by a cold and impetuous wind. Snow and ice were spread over the plain, and produced a cold so intense that the movements of men and animals were nearly retarded. Levesque† has instituted an inquiry respecting the losses in men from excessive cold by the princes and nobles of Russia, who fought with interminable hostility prior to their union with the sovereign monarchy. On the 4th day of October, 1632, the cold became so keen between Montpellier and Beziers that 16 body-guards of Louis XIII, eight Swiss, and thirteen camp-boys were destroyed. Charles XII of Sweden, whom "not want and cold his course" delayed, penetrated into Russia in 1709, and marched to Moscow, regardless of all consequences. The Swedes and Russians could scarcely hold their arms, sinking under an inclement winter and a desolating cold. Unfortunately for this great man, great even in his fall, he saw part of his army perish of misery and hunger amidst the desert and icy places of the Ukraine. Numerous soldiers fell victims to cold during the siege of Asoph under Peter the First—the campaign of Moldavia under Catherine the Second—and that of Persia under Paul the First. In 1719, 7000 Swedes, on their road to besiege Drontheim, expired from insupportable cold amid the snows of the mountains, which separate Sweden from Norway. These, and some other facts, have been very properly dwelt upon by the author, and it is impossible to contemplate them without emotion, since they triumphantly prove that in every age the many have been sacrificed by the few, and that human life has ever been accounted as nothing when it could be made subservient to criminal ambition.

Baron Larrey, surgeon in chief to the French army in the Russian campaign, published a valuable history of it, during the year 1817, in a work entitled, "Memoires de Chirurgie Militaire, et Campagnes du Baron D. J. Larrey, &c. (vol. IV.) and of which a faithful and comprehensive analysis was pre-

---

* In Vita Alexandri Magni, Latin and French Edition, 1716.
† History of Russia.

sented in a former number* of this Journal. But in that volume comparatively little was written concerning cold; and as the present is professedly devoted to the consideration of the effects and properties of it, we, by treading in the path of analytic detail, are enabled to offer a novel and interesting subject for the gratification of our readers. More than twenty years have elapsed since any systematic work on cold was printed in this country, yet several have recently appeared in France and Germany, and the productions of Kern and Lombard, of Percy, Tanchon, and Brandis, are well known. A writer, whom we need not name, promulgated, with all the confidence which accompanies self-conceit, the following theory of cold.—"Heat is a stimulus; cold is the abstraction of heat; therefore, cold is the abstraction of stimulus, or is a sedative." Is this the language of a philosophical mind? is it thus great and influential truths are to be elicited? Miserable indeed, would philosophy be, and science for ever degraded in an age, which could produce a single convert to such a theory. We shall best expose it by adopting the words of an author, whose opinions on the subject of cold are, and always have been rational. To this we might oppose another theory, equally syllogistic,† and nearer the truth; free caloric is a stimulus; cold is the sensation excited by the passage of free caloric out of the body; therefore, cold is a stimulus. But, in fact, the action of cold is by no means so simple. It is complicated, and varies according to its intensity, duration, and the state of the system to which it is applied. It acts at first as a stimulant, in exciting sensation; then as a tonic, in condensing the living fibre; and, lastly, however paradoxical it may seem, as a sedative, by preventing that distribution of blood into the minute and ultimate vessels, which is necessary for the existence of sensibility and irritability, and also by the abstraction of the stimulus of heat.

The preface of M. Beaupré is so brief and modest as to deserve insertion in the language of his translator, and it is only an act of justice towards him, since it will best state what his opportunities have been, and explain what were his intentions in taking up his pen.

"I had scarcely conceived the design of examining the influence of cold on the animal economy, when the numerous difficulties that surround this question in Physiology and Therapeutics, which has been,

* No. 6, September 1821, Old Analytical Series.

† How unlike what Scaliger wrote of Varro,—"qui vere ac μεθοδιχως philosophatus sit."—*Scaligerana prima, p.* 144.

and still is, the subject of so much disputation, presented themselves to my mind. I foresaw I should have to reconcile opposite opinions, without deviating from the principles of sound medical doctrine. Nothing more was required to make me hesitate about attempting the subject; but I had had manifold opportunity of observing the action and properties of cold; and though, while occupied in professional attendance on soldiers, in climates of very various temperatures, I had in various circumstances made use of cold applications with success, I confess still, that for want of analytical examination, I had not always been able to explain exactly their physical effects, or manner of acting on the animal economy. The Russian campaign, the sight of the sufferings originating from cold, of which I have been unhappily witness, —what I have myself endured,—what I have read, in short, have induced me to reduce to writing various reflections on this agent, which exercises such great influence over man in health and disease, and to add to them a number of facts from my own observation, or scattered here and there, and which became necessary as a basis for reasonings, and source of useful inferences. It has been my object to make a practically useful work, and to rectify the conclusion still adhered to by some physicians, who, from over-confidence in Brunonian principles, persist in recognizing in cold only an absolutely debilitating property. This double motive gives me some claims on the indulgence of the reader." —*Preface.*

This work consists of nine chapters, namely, I, *Caloric and heat.* 2, *Cold in general.* 3, *The effects of cold on the animal economy.* 4, *Historical and medical view of the Russian campaign.* 5, *Asphyxia, gangrene, and death from cold.* 6, *The application of cold to the hygeia.* 7, *Therapeutical effects of cold.* 8, *Refrigerants.* 9, *The application of cold to disease;* and to which the respectable translator has added an appendix of 52 pages "to strengthen some positions of the author, which appeared to need farther evidences, or as *riders* on clauses in the original, containing decisions too exclusive and unqualified."—Of the last six chapters we intend to give distinct analyses, and of the first three, obviously least important, the following general summary is attempted, to save space for graver and better matter.—1, Moderate heat, like moderate cold, excites organic action; 2, Heat exalts sensibility, but diminishes contractility: whereas, cold lessens sensibility, and raises contractility; 3, The moderate action of heat and cold is alike conducive to the support of life; their immoderate effects alike injurious, since they annihilate moral and physical power. Extreme heat causes apathy and languor, dissolution of the blood and other fluids, gangrene, and death. Excessive cold chills, benumbs, debilitates, and produces even sphacelus, and loss of life.

4. *Historical and Medical View of the Russian Campaign.*—The historical part is purposely passed over, because it will be found in a preceding journal,* and we studiously, and on every occasion, avoid repetition. The fatigues of marching, atmospherical variations, excessive heat by day and sharp coldness by night, the bad quality of the water that served for drink or the preparation of food, and which was often drawn from swamps and marshes, or rivulets at the bottom of ravines, in which carcases of men and horses were putrifying, abuse of grain spirit, food alternately scarce and plentiful, sometimes good and sometimes bad, an irregular mode of life, deficient distribution of necessaries, which often led to plunder, may be enumerated as causes injurious to the health of the French soldiers, and in consequence many suffered from illness. Diarrhœa, by quick depression of strength, did much mischief, and this was occasioned by impure water, and bread made principally of rye. When bread was wanting, the soldiers fled to flour, and with this flour, already warmed, they mixed broth or water, seasoned with a little salt. With this compound they greedily satisfied their appetites, and concluded with a glass of water, or of pungent empyreumatic grain spirit. This food underwent an acid fermentation in the stomach and intestines, which rendered it indigestible, and gave rise to windy colics, a mucous atony† of the intestinal tube, and an abdominal colliquation that wasted the body. The heifers, goats, sheep, and oxen, belonging to each regiment, sank into contagious disease from neglect and fatigue. Next followed the conflagration of Moscow, where, amidst horrible disorder, beer, wine, and brandy abounded, and of these many soldiers largely drank. Such excesses, after such privations, were debilitating—they multiplied disease, and consigned numbers to

* No. 6, September, 1821. Old Analytical Series.

† This is a singular phrase, and, with some others, must be attributed to the author, or his translator. At a time when there is such an unhappy rage to coin words, to make them, not what they ought to be, the symbols of ideas, but "the concord of sweet sounds," it may be excusable to examine into the meaning of "mucous atony." The obvious meaning is a mucous weakness, yet this, we conceive, is not meant, and we guess it is intended to imply an atony of the mucous membranes. A rhetorician would pronounce such a phrase to be a burlesque *metonomy*, and would reprobate the imitation of it. Those who are responsible for this and similar terms would do well to think of Julius Cæsar, who, in his work "*De Analogia*," which, unfortunately for literature, has not been preserved, recommended authors and public speakers to avoid, as a rock, every unusual word, or unwonted expression.*—*Rev.*

* Vide Aul. Gell. Noctia. Attic. Lib. I. c. 10.

hospitals established in edifices which had escaped destruction. The burning of Moscow, the anticipated haven of luxurious ease, completed the misery of the French army, and many soldiers, alas! too many, were seen sick or wounded, retreating at random, sad, pale, and dejected, begging with tears in their eyes for a morsel of bread. Moscow was evacuated in the middle of October, 1812, and a retreat was commenced under hardships that would have appalled the stoutest heart. The autumn had been mild, the fine weather seemed prolonged, and the frost was delayed to mock human hopes. From the 20th to the 25th day of October the cold became intense, and the rigor of winter began to press upon the retreating army. Food speedily diminished in quantity, and horses died on the road from exhaustion along with unhappy soldiers. Unrelenting cold, want of food, and ravenous hunger imperiously forced soldiers, officers, physicians, commissaries, and other functionaries to eat the flesh of horses, which lay in the roads, fields, and ravines. Those who were strongly impelled by hunger ate that flesh raw, but usually it was roasted at the bivouack fire, which only rendered it harder and drier. The soldiers had no longer strengthening drinks:—coffee a little supported the officers. The ice was broken to obtain water to dress horse flesh, or the refuse of vegetables and aliments that the strictest search, and the most cunning marauding could procure. Discipline was relaxed, licentiousness arose from want of food, and numerous soldiers wandered into the plains, and perished by cold and hunger, the sword of the Cossack, or the vengeance of the incensed peasant. Encamped on a frozen soil, sometimes without fuel to light fires, these wretched men, whose clothes were torn or destroyed, arrayed themselves in the most fantastic dresses to obviate the effects of the most piercing cold. The dying and the dead were plundered of their coverings, the petty distinctions of military rank were forgotten, and all were equal when misery and privation reigned. Still, a great number, like desperate pilots steering with shattered sails—men, whose all was courage, stemmed an almost overwhelming torrent, yet even they, to whom victory was familiar and death a blessing, sank in strength:—the skin was dry, filthy, earthy, contracted; the face was care-worn; and a long beard seemed to denote wickedness and dirtiness. Many fell victims to a frightful bulimia. Men quickly died on the road, or slowly expired, for neither assistance nor asylum could be obtained. Melancholy and nostalgia did infinite mischief; they either prepared or accelerated destruction. Along the road, or in the neighbouring ditches and fields, human bodies were to be seen in fives, tens, fifteens, and twenties, which had perished during the night. Smolensko the army reached early in November, but affording no succour, it was abandoned in a few days, and here again the wounded were consigned to the implacable elements, and reposed on snow which was stained with their blood. Horse-flesh was a never failing resource to the surviving and proceeding troops, and they even became nice as to particular parts, for the brain, tongue, and liver were ever considered to be most tender and delicate. Many individuals had their hands, feet, and ears frozen, and happy were they that escaped the loss of an entire member. Some remained, mortally seized by cold, when obliged to stop to satisfy pressing necessities. Wilna, already exhausted, furnished few resources. The cold was then most rigorous, for early in December it ranged from 9° to 20°. United in one city, the prey of disease and alarm, an immense number of feeble beings, and of expiring sick and wounded, were stretched on the streets and squares, without fire or succour. The private houses and hospitals were filled with about 16,000 patients, and thus did the asylums of humanity become the tombs of mortality. The remains of the army, attacked and harrassed by the enemy, were obliged precipitately to quit Wilna, and marched between rows of frozen corpses, or abandoned persons, who had died unknown and unregarded. Towards the middle of December, 25 to 30,000 men, fragments of a once formidable army, repassed the Niemen. On the public place of Krasnoe, in the villages, through which prisoners of war marched, and on the road to Kursk, heaps of frozen corpses recalled to the mind the verses of Corneille, depicting the battle of Pharsalia.

"Ces montagnes de morts privés d'honneurs funebres."

The victims of cold and misery were seen walking, insensible and unconscious, and when human nature, too hardly taxed, could do no more, they sank never again to rise. The danger of stopping was imminent:—safety was only to be had in motion and stimulus. The pulse of those, whose fate was sealed, was scarcely perceptible. Respiration, very infrequent, and hardly sensible in some, was attended, in others, by complaints and groans. Now the eye was open, dull, fixed, wild, and the brain was seized by quiet delirium;—next, the eye was red, announcing transient excitement of the brain, with more marked delirium. Some stammered incoherent words;—many had a convulsive laugh. Blood flowed from the nose and ears of several soldiers, who agitated their limbs as if for a specific object. Numbers, overpowered by cold, uncovered their chests, and shook their arms like those "labouring under deaf delirium in an ataxic fever." Here they no longer felt a desire for food, and the spastic pressure of the lower against the upper jaw was almost constant, and increased with the progressive effects of that cold, which caused torpor and death. In this manner did thousands perish, and ultimate sickness was nearly the universal lot of those that escaped. A number of soldiers, more or less seriously injured by cold, during the year 1813, filled the hospitals of Poland, Prussia, and Germany. From the shores of the Niemen to the banks of the Rhine the fragments of an army, desolated by cold and misery the most appalling, were to be recognized. Many, whose climax of sufferings was not yet complete, distributed themselves into the hospitals on this side of the Rhine, and even as far as the south of France, to undergo various extirpations, incisions, and amputations. Mutilations of hands and feet, loss of the nose or of the ear, weakness of sight, deafness, neuralgia, rheumatism, palsies, chronic diarrhœa, and pectoral affections attested the calamities resulting from the expedition into Russia. The power of cold over the fauces and trachea, as well as the voice, not particularly noticed by the author, has been finely expressed by a writer who, for his talent and poetry, deserves to be better known, and more read.

> "Ægrescunt teneræ fauces, quum frigoris atri
> Vis subiit, vel quum ventis agitabilis aër
> Vertitur, atque ipsas flatus gravis inficit auras,
> Vel rabidus clamor fracto quum forte sonore
> Planum radit iter. Sic est Hortensius olim
> Absumptus: caussis etenim confectus agendis
> Obticuit, quum vox, domino vivente, periret,
> Et nondum exstincti moreretur lingua diserti."
>
> *Seren. Samonicus, De Medicina,* c. 15.

5. *Asphyxia, Gangrene, and Death from Cold.* Few words have been used more loosely by the profession than the word asphyxia. This term from α, priv. and σφυξις, a pulse, simply signifies the absence of the pulse, but is now applied to every apparent loss of vitality. By the present author it is employed to denote "partial congelation" from cold, and it is to be understood in this sense in the pages of this analysis. The states about to be considered are produced by an abstraction of caloric, and stupefaction of the vital powers, resulting from an excessive or prolonged application of cold, either to a part, or the whole of the human frame. The insidious effects of this agent may extend from the slightest numbness to the suspension and abolition of every motion and feeling. Individuals are generally least or most obnoxious to it in proportion to their powers of resistance. Soldiers, whose dress is little prepared for exigencies, often have their ears and fingers attacked by asphyxia and mortification, while troopers, frequently and even unknowingly, have their toes and feet frozen. Sentinels, victims of an icy night, have been found stiff and motionless. Repose or inaction is dangerous in cold weather. If a sleeping person be exposed, and insufficiently covered, cold soon seizes him, because the vital motions are slower, and favourable re-action is feeble and powerless. Asphyxia and gangrene have supervened upon transition from a high to a very low temperature, or from sudden variations as Larrey has experienced. Moderate stimulus is beneficial to him that keeps in motion, but detrimental if he stop and sleep. Asphyxia* from cold is divided into *partial* and *general*. The local is the effect of cold on the nose, cheeks, lips, chin, ears, feet, and hands; parts far removed from the heart, and whose vitality is consequently less active. *Partial* asphyxia presents various phenomena in its progress, which furnish grounds for a distinction of three degrees.—*First Degree.*—The general action is more manifest on one of the parts exposed. The alteration of the vital properties commences with painful formication. The skin of the extremities of the fingers, nose, ears, &c. after having been for a long time red, hard, and affected with painful prickings, grows pale; its temperature and sensibility diminish; its vitality seems entirely extinct. Local numbness gradually succeeds a disagreeable sensation, of which the subject is glad to be relieved, and thus he is unconscious of a change from bad to worse. Yet this unhappy change may be prevented by frictions with snow, for the points of the nose, &c. at this critical juncture are of a horn white, and resemble the colour of old wax.—*Second Degree.*—After entire cessation of pain the part remains cold and insensible; sometimes phlyctenæ arise; and occasionally the alteration of colour in the skin, which is livid and blackish, evinces from the beginning, mortification. In both of these degrees, re-action infallibly supervenes; and it is announced by lively

* The reader will be pleased to recollect the sense in which this word is employed by the French author. Literally it means a want of pulse, more extendedly an apparent deprivation of life; M. Beaupré uses it "to express partial congelation, only because modern writers have "shewn him the example." Such an example would have been "more honoured in the breach than the observance."—*Rev.*

smart pain, and redness of the skin. When moderate, it is salutary, but it is not without danger when too impetuous, or concentrating its action on a particular spot. On the energetic return of sensibility and contractility, no matter how induced since the effect is the same, the blood and humours flow into the dilating tissues, and occasion a circumscribed distention. An insupportable formication and itching arise in the whole part; sometimes the pain calls forth complaints and cries; and ardent inflammatory action is established along with sharp and biting heat. When the skin, at the highest re-action, preserves an equal redness, the fear of gangrene is less than when it assumes a livid, or violet marble colour. Here the epidermis rises in different places, forms brown or blackish vesicles, which are filled with a serous, bloody, ichorous, or yellowish fluid, whose presence increases the disagreeable and obtuse sensation that is experienced. This disorder is not confined to simple elevation, and desquamation of the cuticle in layers.—It extends into the interior, and constitutes the second degree of local asphyxia since the cold destroys the skin, tendons, or aponeuroses, while eschars of various extent are produced along with an inflammatory circle.—*Third Degree.* This is sphacelus. If to paleness and sensibility there be added a total cessation of organic action, and a feeling of very great weight; if an easy separation of the epidermis disclose a livid marble colour of the cutis, and if to the flaccidity of the muscular fibre, which is insensible to stimulants, there succeeds a putrid exhalation, no doubt can be entertained of the complete sphacelus of the part implicated. *Treatment.* The *contraria contrariis medentur* would here be very dangerous in practice. In very slight numbness, a gentle and graduated heat to dissipate it, or moderate frictions with woollen cloth may be employed without inconvenience:—But when there exist complete stupor, and suspension of all motion, heat is unsuitable, since it would be sufficient to cause rapid gangrene by a sudden change of local condition. The true method consists in slowly exciting reaction, in gently recalling the vital properties, and preventing their too rapid restoration. It is proper to begin with efficacious means on the confines of the asphyxiated part, and afterwards they are to be used generally. Frictions with snow,* or pounded ice, are best:—when

* The general practice of applying snow to frost-bitten parts, appears to be contrary to the observation of an enterprizing navigator, who must have had considerable personal knowledge of the powers and effects of intense cold.—"The Eskimaux have a very effectual way of restoring the circulation, which is by laying a warm hand on the place affected. We had always been they seem to do good, the transition should be to water successively less cold, and after the part has become sensible lukewarm water is to be applied, and ultimately warm water under prudent restrictions. If the violet, or black spots have disappeared, if the affected spot be soft, supple, and red, we must confine ourselves to simple dry frictions with flannel. Where re-action is feeble, where insensibility shows itself with an atony almost œdematous, and where sphacelus threatens, it is allowable to employ "embrocations prepared from oak-bark, cinchona, mustard, aromatic plants, &c. and to which wine, alcohol, myrrh, and camphor may be added." If, at the end of a few hours, the skin have not lost its livid, or violet hue, it is to be scarified, and treated with the topical means just enumerated. Turpentine has been regarded as an advantageous remedy for dissipating numbness from cold. Internal excitants may sometimes be useful, and they shall be noticed under *general* asphyxia. Gangrene once established, whether primary or consecutive, only demands ordinary management, and, according to strength and means, nature, sooner or later, makes a boundary between the dead and living tissues.—*General* asphyxia extends at once over the whole machine, and exhibits the image of perfect death; yet persons, found senseless and benumbed, have been brought to life after a lapse of twenty-four, or forty-eight hours. Man re-acts against a rigorous and immoderate cold as long as his strength and courage will allow, but re-action has its limits, and a moment arrives when the powers of the vital principle are exhausted. Then, and not till then, the faculties, physical and moral, seem as if enchained, and at length abandon that body, which they have so stoutly clung to under every disadvantage, to the progressive and always increasing power of cold. Shiverings, puckerings, paleness, coldness of the skin, livid spots, and "muscular flutterings," indicate the shock which is given to the vital forces. The subject feels syncope approaching; his stiff muscles contract irregularly; his body bends and shrinks; his limbs are half-bent; sometimes lassitude invites him to repose;—occasionally a feeling of weight and general numbness retards his steps; his knees grow weak, and he either lies down or falls. He then experiences an invincible propensity to sleep; every thing around him appears strange; his senses are confused, his mind

---

accustomed to rub the spot with snow, which frequently caused irritation, and left the part so tender as to render it extremely susceptible of other attacks."—*Capt. Lyon's Private Journal, &c. &c. London*, 1824, *Octavo, page* 124.—*Rev.*

dull, his ideas disturbed, he stammers and raves, and if he be free from suffering he is often not so from agitation. Now he cannot be roused, he entreats to be permitted to sleep;—he slumbers; remote parts become cool; respiration, at first interrupted, is slow; the contractions of the heart are feeble, quick, hard, irregular, and sometimes painful; the pulse is progressively smaller; the central heat is extinguished; the brain stupefied; the pupils dilated; and, finally, a deep and mortal coma is inevitable unless fortunate succour arrive.—*Treatment.* The body is neither to be transported into a heated place, nor immediately are warm substances to be applied; for strong re-action would, perhaps, exhaust the remaining vitality. Again, the rapid return of circulation to the surface, and the dilatation of the tissues, owing to sudden transition from cold and condensed to warm and rarefied air, produce shooting pains, dyspnœa, suffocation, and even death itself. Numerous are the instances of travellers who, greatly suffering from cold, have perished from immediate exposure to a large fire, whilst those that have taken opposite means have been preserved. No state of asphyxia, however apparently complete, can justify the neglect of aids appropriate to the excitation of vitality. Individuals long buried under snow oftener recover than those do who have been exposed to the air. Curious cases of such recoveries are to be found in the Bibliotheca Britannica, the old Journal de Médecine, and the Memoirs of the Academy of Stockholm. The body should be quickly stripped, laid on a hard surface, and placed where there is no current of air, and where the temperature is a little above that of the atmosphere. Frictions with some exciting tincture are to be made on the præcordial region and umbilicus, and followed by the application of warm coverings. Afterwards the use of snow, iced water, and water successively less cold is to be employed. This first operation ought to continue nearly fifteen minutes; next, water a little warmed, and, ultimately, luke-warm, and hot water. When circulation and respiration are sensibly restored, when the muscles lose something of their inflexibility, and a little heat is manifested, the body must be quickly wiped with dry linen, subjected to dry frictions with flannel, and wrapped in a woollen blanket. Irritation of the nostrils, rude frictions with a brush dipped in vinegar on the hands and feet, brandy, wine, enemata, rich broths, and medical stimuli claim attention. When the vital heat is uniformly established, and the chest dilates itself well, and the motions of the heart are strong and regular, exciting drinks must be interdicted to guard against morbid re-action, fever, or inflammation. A slight moisture should be maintained on the skin, and the first solid food ought to be light and restorative. Cold produces the fatal effects enumerated whether above or below 0°.—Moderate cold continued causes the same consequences as severe cold of short duration. Cold sometimes acts like opium, inflicts a deadly stroke on contractility and sensibility, directly benumbs the central system, and the irritability of the heart. Prosper Alpinus, in his work, "*De Presagienda Vita et Morte,*" has promulgated similar opinions. As the action of cold is commonly slow, and the exposure of a few hours duration produces death, the calibre of the vessels diminishes, and repels the blood towards the cavities of the head, thorax, and abdomen. The consequences are an obstruction in the pulmonary circulation, and venous system of the brain, which disturbs the functions of that organ, and induces somnolency. These statements are corroborated by the flowing of blood from the nose and ears and lungs, unnatural redness of the viscera, engorgements of the cerebral vessels, and sanguineous effusions. It would seem that cold sometimes causes excitation of the brain which may prove mortal. Battie* saw a man who became maniacal after exposure to very rigorous cold, and Boerhaave† mentions a coachman, that died of cold, in whom there was inflammation of the brain, particularly of the right hemisphere, which adhered to the dura mater by a false membrane.

6. *The Application of Cold to Hygeia.* Cold acts on man, 1st, By a tonic property direct, derived from vital re-action; 2dly, By a tonic property indirect, resulting from abstraction of stimulus, or prevention of excessive diffusion and dissipation of organic activity. The former occurs in winter, and the latter in summer. In regard to therapeutics, cold may be considered under the heads of air, bathing, drinks, and aliments. Here the author's observations are judicious, but well known, and need not be farther entered into, since they are only subsidiary to practical medicine, and to the common occasions of life. The effects of cold air and bathing, cold drinks and aliments cannot be deemed desiderated information in an age so enlightened.

7. *Therapeutical Properties of Cold.* Cold, as a curative agent, has fixed the attention of ancient and modern physicians. Melampus, who, at his return from Egypt, introduced the use

---

* See his Aphorisms.

† Comment. Lipsiens. vol. I. pars I, p. 232.

of the cold bath amongst the Greeks, may be named as the first, and likewise Erasistratus, Musa, Hippocrates, Avicenna, Celsus, and Galen. An "attentive and elaborate analysis has led" the author to distinguish and determine upon seven therapeutical properties of cold; namely, 1. A refrigerating property; 2. An exciting; 3. A sedative; 4. An astringent; 5. A tonic; 6. A debilitating; and, 7. A perturbing property. —1. *Refrigerating property.* This is sufficiently shown during fevers and inflammations, where the skin is hot and dry, the eyes sparkling, the lips parched, the breath hot and fetid, the pulse hard, the thirst intense, and where cold, internally and externally administered, acts like a charm, and induces perspiration, tranquillity, and sleep.—2. *Exciting property.* From this, animal and organic life receive a shock that renders them more active, and occasionally overcomes insensibility and torpor—*e. g.* syncope, asphyxia, the hysteric paroxysm, cold affusions to the head, or ice to the eyes, forehead, temples, or back of the neck. Thus the washing of the lower half of the body with iced water, frictions with snow or ice on the sacrum, or pubes and perineum, have been successful in palsy of the bladder, and incontinence of urine from debility. The washing of the abdomen with cold water excites the peristaltic motion of the intestines, and produces evacuations, while the same effects have resulted from cold water thrown on the belly and limbs.—3. *Sedative property.* Cold enjoys a sedative effect very different from its debilitating one, for the first operates by merely diminishing excessive sensibility, while the second is exercised on, and destroys all, the vital properties. A few simple facts will prove this sedative power of cold. The exaltation of sensibility in the pharynx, œsophagus, and stomach, caused by pungent food and drinks, &c. is allayed by cold water, as well as the keen thirst of wounded persons after operations, and which is only the result of nervous irritation. An enema of cold water lulls the feeling of pungent heat, and the painful gripings of hypercatharsis. The application of iced water subdues spasm. In diseases marked by increased sensibility cold acts like the most potent narcotics; and the bath and fomentations are the most useful forms.—4th, *Astringent property.* This is evinced by cold producing an internal fibrous contraction in living tissues. This occurs only in the sick, where debility is not considerable. The employment of cold as an astringent removes looseness of the skin, cures or relieves relaxation of the sacro-sciatic ligaments, emphysema, relaxations, particularly of the scrotum, penis, and labia, prolapsed vagina, uterus, and rectum. Simple washing with cold water arrests slight hæmorrhages, and benefits thrombus, and bloody tumours from contusion.—5, *Tonic property*. The body or a part is strengthened by cold applications which provoke re-action. Cold overcomes original weakness, and invigorates the frame. The number of diseases benefited by the agency of cold is well known to the most cursory observer.—6, *Debilitating property*. Cold is one of the most potent debilitants in therapeutics. Abstraction of caloric, universal constrictive spasm, diminution of sensibility and contractility to the last degree, are its principal destructive efforts. Yet still cold as a debilitant may be advantageously used in one class of diseases, and the ends to be attained by it are, 1st. To diminish excessive vital action, local and general, to lower the excitation of all the vital properties, and to restore them to their accustomed healthy condition. 2dly, To reduce them to a suitable degree that the disorder may follow a safe and regular march. 3dly, To produce a judicious exhaustion of the constitutional powers to prevent their extraordinary development, or a recurrence of their probable exaltation. Cold applications, improperly applied, act externally and internally;—first, they weaken the part, then, the effect extends, by continuity and sympathy, over the whole frame, and, besides other obvious mischiefs, they sometimes cause inflammation to terminate in gangrene.—7. *Perturbing property*. This depends on sensation and brisk spasm. The strong and unusual impression of cold excites general surprise, and produces over the whole sensitive system a sudden shock which modifies its condition. Hence its capability of breaking through the continuity or periodicity of certain nervous movements; and in this way it is to be considered as antispasmodic, whether acting immediately or by sympathy. In the latter case, the perturbation is a revulsive excitement, and destroys the movements existing elsewhere. Spasms yield to sudden cold:—cold water removes hiccup:—iced water enemata have subdued convulsions:—ices have cured hysterical women of palpitations, and imminent suffocations. Affusion of cold water on the head has been beneficial in certain cases of vertigo, and obstinate head-achs. Immersion by surprise has successfully treated intermitting mania. Thus are cold applications useful in active hæmorrhages that depend essentially on the nervous system.

8. *Refrigerants*. Every substance, solid or fluid, simple or mixed, which, when applied to any surface, whether internal or external, produces all the effects of cold, is denominated refrigerant or frigorific. Under that general name shall be included; 1, *Cold air.* The use of this is manifest in inflammatory and febrile diseases as well as for the purposes of health. —2, *Common water.* This is the easiest way of applying cold, and its temperature can readily be graduated. Between 65° and 80° water is cool: it is called icy when extremely low and little removed from the freezing point. Water may be brought to this state by a sufficient quantity of ice.—3, *Sea-water*, which is colder in proportion than fresh.—4, *Cold mineral waters*, natural or artificial, chalybeate, saline, sulphureous, or acidulous.—5, *Snow or ice.* They are exhibited by apposition, friction, or as epithems, until completely liquefied. The ice should be pounded, and inclosed, like snow, in a linen bag or bladder three-fourths full. The degree of cold may be increased by the addition of three parts of kitchen salt, or three of sal ammoniac to eight parts of ice. Ice can be introduced into certain cavities by deglutition, or other means.—6, *Mixture of neutral salts.* Desiccated muriate of soda, muriate of ammonia, nitrate of potass, the sulphate, phosphate and carbonate of soda, and desiccated muriate of lime, are convenient for the production of cold. A bag of fine linen is to be filled with two of these salts quickly mixed and moistened with water. An intense cold is made by the admixture also of nitric or sulphuric acid with snow.—7, *Mercury.* This metal causes a lively feeling of cold, owing to the rapidity with which, like many other bodies, it abstracts caloric, and is, therefore, available to practical medicine.—8, *Ether.* Its rapid evaporation quickly carries away the principle of heat.—9, *Herbs.* Cold is applied, internally and externally, in different forms. The following are the principal:—1, *Aliments.*—2, *Cold drinks.*—3, *Ices.*—4, *Cold bath.*—5, *Immersion.*—6, *Pumping or douche.*—7, *Affusion.*—8, *Aspersion.*—9, *Fomentation.*—10, *Embrocation.*—11, *Lotion or washing.* Subjects like these are familiar to every student, and render comment superfluous.

9. *The Application of Cold to Disease.* This is the longest chapter in the present work, and we hesitate not to say the least interesting and important, for it only names, and comments on the various diseases, in which cold, in one form or other, either has, or is supposed to have, a beneficial operation. The information which such a chapter can impart will not be required by any intelligent reader, and to him that is the reverse it could be of little use, but as we have not yet indulged in a single extract, excepting the preface, it may be pardonable to offer a short one from this chapter, which will answer the double purpose of displaying the manner in which M. Beaupré treats a medical subject, and indicating the power that his translator, Dr. Clendinning, possesses over the English language.

"Hæmoptysis in an hæmorrhagy in which the application of cold offers, at once, probability of success and hazard of mischief. The irritation which causes and sometimes sustains it, may be exasperated and bring on pneumonia. However, when hæmoptysis is considerable, and menaces the life of the patient, we must without delay recur to means of arresting it. Of all, cold is the quickest and most effectual; it merely requires prudence in its application. The cases inserted by Renard in the Journ. de Med. 1771, show that he has repeatedly cured hæmoptysis, by having pieces of ice held in the mouth, and afterwards swallowed, and even by applying pounded ice on the chest and neck. Gervasius, in his Treatise on the Use of Water, recommends the application of sponges dipped in iced water, to the chest. M. Gallereux, physician at Tonnere, has inserted, in Sedillot's Journal, a very interesting case of hæmoptysis cured by cold drinks, in a weakly valetudinary of sixty. Besides the symptoms peculiar to hæmorrhage, which was abundant and alarming, there were lypothymia, pulse irregular and intermittent, subsultus tendinum. Prescription: pounded ice with sugar internally; bags of ice on the chest; ice in the hands; iced water on the arms; very low temperature of chamber; doors and windows open: the symptoms ceased and recurred three times; the recovery was complete. M. Sedillot subjoins to that case a fact from his own practice, showing the success he has obtained from cold in an analogous case. He had the patient constantly ventilated, and ordered compresses steeped in iced vinegar, or a bladder of pounded ice to the chest.

"Pediluvia by immersion simply, or affusion on the lumbar region, may also be employed. Iced drinks are given, rendered slightly acid or mucilaginous, or in an emulsion, and sweetened to the taste of the sick. When De Haen failed of arresting hæmoptysis by the means pointed out, he prescribed cold water internally. He states, that in one case he premised bleeding, and the patient recovered." 264.

We will conclude this analysis by recording on this page the opinions of Mr. Brodie, concerning the production of animal heat, and the operation of cold. Animal heat is in some way or other dependent upon the integrity of the functions of the nervous system; and, therefore, the absolute degree of cold which an animal can bear with impunity will, *cæteris paribus*, be determined by his capabilities of generating heat. Individuals long exposed to intense cold do not endure a painful state, for they gradually lose their sensibility, become drowsy, and die as if by the effects of an opiate. Cold then acts in the following order.

1. It lessens the irritability, and impairs the functions of the whole nervous system.

2. It weakens the contractile powers of the muscles.

3. It causes contraction of the capillaries, and thus diminishes the superficial circulation, and stops the cutaneous secretion.

4. It probably destroys the principle of vitality equally in every part, and does not exclusively disturb the functions of any particular organ.

Those positions have been confirmed by experiment. Dr. Chassat states, that in an animal immersed in a cold bath, death may take place at 79° Fahr. (26 *Centig.*) although it may be sometimes cooled as low as 69° (17 *Cent.*) before the animal dies;—but, *cæteris paribus*, the animal dies sooner as the cooling is more rapid. After death the blood is generally florid in the aorta, so that the animal does not die of suffocation. The heart occasionally contracts feebly after the muscular irritability of the limbs and intestines is nearly destroyed. The cerebral veins contain but little blood, and the ventricles only a small portion of fluid. Chassat's experiments coincide with Brodie's, for he uniformly found the heart much distended with blood, and, as in syncope, scarlet blood occupied the left side of it. Invariably the heart ceased to contract before the diaphragm.*—We have added the preceding information because it fills an obvious chasm in the present performance, and which chasm has not been obviated by the translator. We will still farther explain what we mean. When a human being perishes from cold, it is interesting to ascertain how he dies, and what particular functions necessary to life first sink under that destructive agent. Do the functions of the lungs, or does the action of the heart stop first?—If the functions of the lungs, then, the sufferer expires, as they do under strangulation, drowning, and the inhalation of noxious airs; and the left side of the heart and aorta will display black and unoxygenized blood, which is inadequate to the prolonged stimulation of these parts, and injurious to the brain. But if the action of the heart fail first, as it does in syncope, the left side of that organ and its artery will contain scarlet or oxygenized blood. In the former instance inflation ofthe lungs would be a judicious step:—in the latter a most absurd proceeding. In the matter now under consideration we may, perhaps, safely conclude that intense cold slowly, yet certainly, affects the whole nervous system; that, in consequence, the irritability of the heart, and

* Paris and Fonblanque, Medical Jurisprudence, vol. II. p. 61—2.

of every other muscular part, gradually fails; and that, finally, life departs with a simultaneous cessation of every function on which its continuance depends.

Our opinion of the present work is decidedly favourable. The author appears to be a cautious, sensible, and well informed man; and he shows an acquaintance with general medical literature, which is truly creditable to one who has, probably, passed his life either in the dissipations of a garrison, or amidst the hardships of war. Undoubtedly the work has its faults;—the arrangement is objectionable; the chapters are too numerous; there are frequent repetitions, and words are employed in a loose, ambiguous, and even unauthorized acceptation.

The translator now comes under our notice. From indirect evidence we judge him to be a native of Ireland, who has recently graduated in Edinburgh, and who there read, translated, and gave to the world this publication. Not having even seen the original we cannot express any opinion respecting the fidelity of the translation. We are told the translation is a close one, and by the word "close" it is presumed the translator meant that he had followed his author word for word. This, and only this, he could mean, since the language is incorrect, harsh, diffuse, and abounds with colloquial phrases, which are seldom heard in good conversation. The appendix represents Dr. Clendinning in a respectable light; it indicates him to be a sincere lover of his profession, and an attentive reader of its varied literature. We advise him to cultivate the elegancies of the English language, to prefer sound opinions to futile hypotheses, to bestow his praise with rigid discrimination, and to scorn flippant injustice whenever it is directed against a departed man of genius.

